# IL-1 Fragment Modulates Immune Response Elicited by Recombinant *Bacillus subtilis* Spores Presenting an Antigen/Adjuvant Chimeric Protein

**DOI:** 10.1007/s12033-018-0117-0

**Published:** 2018-09-03

**Authors:** Wojciech Potocki, Alessandro Negri, Grażyna Peszyńska-Sularz, Krzysztof Hinc, Michał Obuchowski, Adam Iwanicki

**Affiliations:** 1 Department of Medical Biotechnology, Intercollegiate Faculty of Biotechnology UG-MUG, University of Gdańsk, Gdańsk, Poland; 2 Tri-City Animal Laboratory, Medical University of Gdańsk, Gdańsk, Poland; 3 Department of Medical Biotechnology, Intercollegiate Faculty of Biotechnology UG-MUG, Medical University of Gdańsk, Gdańsk, Poland; 4 Department of Microbiology, Faculty of Biology, University of Gdańsk, Gdańsk, Poland

**Keywords:** *Bacillus subtilis*, Recombinant spores, Mucosal immunization, IL-1, FliD, *Clostridium difficile*

## Abstract

Mucosal immunizations are convenient ways of vaccination, which do not require any trained personnel for administration. One of the major challenges for developing an effective mucosal vaccine is finding appropriate adjuvant. *Bacillus subtilis* endospores have been shown to help solving these obstacles while serving as a platform for presentation of both, antigens and adjuvants. In this study, we have successfully designed and constructed recombinant spores displaying an antigen/adjuvant chimeric protein. We have used a fragment of *Clostridium difficile* flagellar cap FliD protein as antigen and VQGEESNDK peptide, a fragment of human IL-1β, as adjuvant. Recombinant spores presenting FliD were able to elicit immune response in orally immunized mice which could be evaluated by detection of FliD-specific IgA antibodies in feces of immunized animals. Moreover, the presence of IL-1β fragment significantly changed characteristics of elicited immune response. Obtained results show that recombinant spores presenting an antigen/adjuvant chimeric protein exhibit both properties in mucosal immunization of mice. Moreover, IL-1β fragment could serve as valuable adjuvant in *B. subtilis* spore-based mucosal vaccines.

**Electronic supplementary material:**

The online version of this article (10.1007/s12033-018-0117-0) contains supplementary material, which is available to authorized users.

## Introduction

The technology of heterologous proteins display on surface of *Bacillus subtilis* spores has been used in different applications since its invention almost 2 decades ago [1]. *B. subtilis* spores have been used for presentation of enzymes, fluorescent proteins, peptides, and antigens (reviewed in [2]). Two main approaches to spore surface display have been developed. First, the recombinant one, requires modification of *B. subtilis* genome to express a passenger protein in fusion with a spore coat protein enabling its incorporation into the forming spore coat. Second approach is based on the adsorption technique and enables presentation of native proteins on surface of spores produced by wild-type strains (reviewed in [3]).

One of the most interesting applications of spores presenting heterologous proteins is the use as carriers of antigens in mucosal vaccines. Mucosal vaccines, despite a number of potential advantages over injectable ones (such as no need of injections and hence no risk of transmitting blood-borne diseases, and easy way of administration), are much less common. Most soluble protein antigens introduced via the mucosal route are poorly immunogenic and induce specific, long-lasting tolerance [4–6]. Moreover, the problems with rapid antigen degradation on the mucosal surfaces and lack of appropriate mucosal adjuvants primarily contribute to their diminished usefulness [7]. The technology of spore surface display seems to be a remedy for some of these drawbacks. *B. subtilis* spores were successfully used to elicit immune response upon mucosal immunization against such pathogens as *C. perfringens* (mice) [8], *C. tetani* (mice) [9], *Clostridium difficile* (hamsters) [10], or rotavirus (mice) [11]. Nonpathogenic status of *B. subtilis*, simplicity of construction of recombinant spores presenting heterologous protein, as well as efficient surface adsorption, combined with easiness of spores’ production and administration make them especially interesting carriers of antigens in mucosal vaccines.

The constantly increasing number of trials to use spores for eliciting mucosal immune response comes along with better understanding of mechanisms of interaction between antigen-presenting spores and the host immune system [12]. Spore-based vaccines have been shown to stimulate both systemic and localized immune responses with balanced Th1/Th2 polarization [13]. Unmodified *B. subtilis* spores can also be used as mucosal adjuvants in some applications [14], nevertheless an efficient immune response usually requires use of strong immunogenic antigens such as bacterial toxins [15]. The efficient immunization can also be obtained by co-administration of antigen-presenting spores and adjuvants [16, 17]. Recently, we have successfully used a combined recombinant and non-recombinant approach to display antigen and adjuvant on single spore [18].

Interleukin 1 (IL-1) is a family of cytokines of key importance for host immunity, involved in development of both immune and inflammatory reactions [19]. The human IL-1β domain in position 163–171 comprising the amino acid residues VQGEESNDK has been shown to possess strong adjuvant activity with lack of inflammation-related effects imposed on immunized organism [20]. It has been used to enhance immune responses elicited by immunization with such proteins as bacterial ferritin and flagellin [21] or tumor antigens [22, 23]. Shorter variants of this peptide not only retained adjuvant activity, but in some cases, their adjuvanticity increased [24].

In this study, we have constructed recombinant spores presenting fragment of *C. difficile* FliD protein fused with VQGEESNDK peptide. The FliD is a flagellar cap protein with strong antigenic properties [25, 26]. We have already used a fragment or the entire FliD protein in our previous studies in which we have shown that it required an adjuvant for eliciting an efficient immune response [18, 27]. To our knowledge, this is the first attempt to display on the spore surface a molecule possessing both antigen and adjuvant properties. Such recombinant spores elicited, in orally immunized mice, the immune response characterized by significantly changed cytokine production pattern suggesting immunomodulatory action of the IL-1 fragment.

## Methods

### Ethics Statement

The experiments involving animals were performed according to the institutional and national guidelines for animal care and use. All protocols were approved by the Committee on the Ethics of Animal Experiments of the Medical University of Gdańsk (Permit Number: 4/2010). The procedures were performed under isoflurane anesthesia, and all efforts were made to minimize suffering.

### Bacterial Strains

Bacterial strains used in the study are listed in Table 1.

**Table 1:** List of strains used in this study

Strain	Relevant genotype	Source or reference
*E. coli*		
DH5α	*fhuA2 lac(del)U169 phoA glnV44 W809 lacZ(del)M15 gyrA96 recA1 relA1endA1 thi-1 hsdR17*	[30]
BL21(DE3)	*fhuA2 [lon] ompT gal (λDE3) [dcm]* Δ*hsdS λDE3* = *λsBamHIo* Δ*EcoRI-B int : : (lacI : : PlacUV5 : : T7 gene1) i21* Δ*nin5*	NEB, USA
EAN07	DH5α pAN07	This work
EWP14	DH5α pWP14	This work
EWP15	BL21(DE3) pWP15	This work
*B. subtilis*		
168	*trpC2*	[28]
BAN03	168 *amyE::cotB-fliD*	[29]
BAN07	168 *amyE::cotG-fliD-IL-1*	This work
BWP14	168 *amyE::cotB-gggeaaakggg-IL-1*	This work

### Construction of Gene Fusions

A 280-bp DNA fragment encoding a fragment of FliD (residues 226–311) was amplified using PCR technique with *C. difficile* strain 630 chromosome as template and primers fliDIL1-F and fliDIL1-R (Table 2), which also contained sequence encoding VQGEESNDK peptide being a fragment of human IL-1β. The PCR product was sequentially digested with *Bam*HI and *Sac*I and cloned in frame to 3′ end of the *cotG* gene in the pDL-CotG plasmid [31], yielding pAN07 plasmid. For the preparation of a plasmid encoding the CotB-GGGEAAAKGGG-IL-1, fusion primers, cotBIL1-F and cotBIL1-R (Table 2), were self-annealed and cloned in frame to 3′ end of the *cotB* gene in the pDL-CotB plasmid [29], The resulting plasmid was named pWP14. As a host for cloning, *Escherichia coli* strain DH5α (Table 1) was used. Bacterial strains were transformed using CaCl_2_-mediated transformation of *E. coli* as previously described [30].

**Table 2:** Details of the primers used in this study

Primer	Sequence (5′–3′)	Restriction enzyme
fliDIL1-F	GCT**GGATCC**ACTAAATCTGCAGTAGTATATGGAAAAAATTTAGAAGCTGATGTAACTGATGAC	*Bam*HI
fliDIL1-R	CGC**GAGCTC**TTATTTGTCGTTTGATTCTTCTCCTTGCACATCTTTTGATTTTTTAGTAGTAAC	*Sac*I
cotBIL1-F	**GATCC**GTGCAAGGAGAAGAATCAAACGACAAAATAA**GAGCT**	*Bam*HI/*Sac*I
cotBIL1-R	**GAGCTC**TTATTTTGTCGTTTGATTCTTCTCCTTGCAC**GGATCC**	*Sac*I/*Bam*HI
cotBIL1his-F	ATAA**GCTAGC**ATGCATCACCATCACCATCACAGCAAGAGGAGAATGAAATATC	*Nhe*I
cotBIL1his-R	ATTA**GGTACC**TTATTTTGTCGTTTGATTCTTC	*Kpn*I
BAN07-seq2-up	TAAAACCGCGCGTACTATGAG	–
BAN07-seq2-dn	CGTACTGTGAGCCAGAGTTGC	–

The recognition sites for the restriction enzymes indicated in the table are shown in bold

### Chromosomal Integration

The pAN07 and pWP14 plasmids were digested with single-cutting restriction enzymes. Linearized DNA was then used for transformation of competent *B. subtilis* strain 168 (Table 1) cells as previously described [31]. Obtained chloramphenicol-resistant colonies resulted from a double-crossover recombination event, in which introduced DNA fragment inserted into the nonessential *amyE* gene of the *B. subtilis* chromosome. Selected antibiotic-resistant clones were tested using PCR technique with chromosomal DNA as template and primers AmyS and AmyA [1]. The selected clones were named BAN07 and BWP14 and kept for further studies.

### Preparation of Chromosomal DNA Fragments for Sequencing

A 901 bp fragment encompassing the *cotG-fliD-IL1* fusion gene was amplified using PCR technique with BAN07 chromosomal DNA as template and primers BAN07-seq2-up and BAN07-seq2-dn (Table 2). The PCR product was purified using ExtractMe DNA clean-up kit (Blirt, Poland) and send to sequencing (Genomed, Poland) along with both primers used for amplification.

### Preparation of Spores

Sporulation was induced by the exhaustion method in Difco sporulation medium (DSM), and the produced spores were purified using lysozyme and salts treatment as described elsewhere [32]. PMSF (0.05 M) was also included to inhibit proteolysis of recombinant proteins. Purified spores were treated at 65 °C for 1 h to kill vegetative cells, which left after purification procedure. The spore suspension was titrated then for determination of CFU/ml and kept at − 20 °C. The estimated yield was 5 × 10^10^ spores per 1 l of DSM culture.

### Spore Coat Proteins Extraction

Spore coat proteins were extracted from a 50 µl of a spores suspension density of 1 × 10^8^ spores per 1 ml with a decoating extraction buffer as described elsewhere [33]. Extracted proteins were assessed for integrity by SDS-PAGE and their concentration in samples was measured by a DC Protein Assay kit (Bio-Rad).

### Western Blotting and Dot-Blot Analyses

Extracted spore coat proteins were separated in 12% SDS-PAGE gels and electrotransferred onto a nitrocellulose filter (Roti-NC; ROTH). Western blottings were performed using standard procedures and visualized by developing with BCIP/NBT according to the manufacturer’s protocol (Fermentas; Thermo Fisher Scientific). Serial dilutions of extracted proteins and purified CotB-IL-1 were used for dot-blot analysis. Filters were visualized by incubation with BCIP/NBT as above, followed by densitometric analysis with ChemiDoc XRS and QuantityOne software (Bio-Rad).

### Immunofluorescence Microscopy

Samples were fixed in the medium as previously described [34] with following modification: spores were suspended in TE buffer (20 mM Tris/HCl pH = 7.5, 10 mM EDTA) containing lysozyme (2 mg/ml) and incubated for 3 min at room temperature. The suspension was washed three times with PBS and the surface of spores was blocked for 30 min by incubation in 3% BSA in PBS. Samples were washed three times and incubated overnight in at 4 °C with mouse anti-FliD antibody. Following incubation spores were washed three times with PBS and incubated overnight with antimouse IgG antibodies conjugated with Cy3 (Jackson ImmunoResearch Laboratories, USA) at 4 °C. After three washes with PBS samples were loaded onto the microscopy slides coated with poly-l-lysine (Sigma, USA). Coverslips were mounted on the microscopy slides and viewed using a Zeiss Axioplan fluorescence microscope with the same exposure time for all samples. Images were captured using a camera connected to the microscope, processed with Corel Photo-Paint software, and saved in TIFF format.

### Coupling IL-1 Fragment with BSA and Antibody Production

Chemically synthesized VQGEESNDK peptide corresponding to 163–171 fragment of human IL-1β was coupled to BSA protein using glutaraldehyde [35]. A solution of 5 mg/ml of the synthetic peptide was prepared in PBS. BSA and peptide were mixed in appropriate molar ratio (1 mol of peptide per 50 amino acids of carrier). Equal volume of 0.2% glutaraldehyde solution in PBS were slowly to the peptide/carrier solution with constant agitation. The mixture was incubated for 1 h, then glycine was added to a final concentration of 200 mM and incubated with agitation for another 1 h. The peptide-BSA conjugate was dialyzed against PBS. Anti-IL-1 antibodies were produced in mice using previously described method [31].

### Purification of CotB-IL-1

The CotB-IL1 expressing plasmid was constructed as follows. A 923 bp DNA fragment coding for CotB-GGGEAAAKGGG-IL-1 fusion was amplified using PCR technique with pWP14 plasmid as template and primers cotBIL1his-F and cotBIL1his-R (Table 2). The PCR product was digested with *Nhe*I and *Kpn*I restriction enzymes and cloned into the pBAD vector (Invitrogene), yielding pWP15 plasmid. Upon verification with restriction analysis the plasmid was used to transform *E. coli* strain BL21 (Table 1). The recombinant strain was used to overproduce CotB-IL1 by induction with 1 mM IPTG. A 38 kDa protein was visualized on a Coomasie-blue stained gel in nonsoluble fraction and purified on Ni-NTA Superflow Agarose (Qiagen) under denaturing conditions. 650 µg of purified CotB-IL1 were obtained from 500 ml of culture.

### Immunizations

Five groups of five mice (female, BALB/c, 8 weeks) were orally immunized by intragastric gavage of suspensions of either spores expressing CotG-FliD (BAN03), CotG-FliD-IL-1 (BAN07), CotB-IL-1 (BWP14) or non-recombinant spores of strain 168 as control. A naive nonimmunized group was also included. Immunization doses contained 1.0 × 10^10^ spores suspended in a volume of 0.2 ml and were administered on days 1, 3, 5, 22, 24, 26, 43, 45, 47. Feces, serum samples, entire gastrointestinal tracts, and spleens were collected after completion of the immunization cycle.

### Extraction of Antibodies Form Gastrointestinal Tracts and Fecal Pellets

Entire gastrointestinal tracts were sliced with the use of a razor blade. Sliced tissues were suspended in 1 ml of PBS containing 2% saponin, protease inhibitors, and 0.1% BSA. Suspensions were incubated overnight at 4 °C with agitation and then centrifuged (5000×*g*, 30 min, 4 °C). Supernatants were transferred into new tubes and centrifuged for a second time (16,000×*g*, 10 min, 4 °C). Final supernatants were collected, and sodium azide was added (3 mM final concentration) as a preserving agent. Five fecal pellets per immunization group were suspended in 500 µl of PBS containing 1% BSA, protease inhibitors, 25 mM EDTA, 10% glycerol, and incubated overnight at 4 °C with agitation. The suspensions were centrifuged (16,000×*g*, 10 min, 4 °C), and supernatants were collected, and sodium azide was added (3 mM final concentration). All extracts were stored at 4 °C.

### Indirect ELISA for Detection of Antigen-Specific Antibodies

FliD-specific antibodies in sera, saponin extracts of gastrointestinal tracts, and feces of immunized animals were detected as previously described [36]. FliD-specific IgG antibodies in sera were expressed as reciprocal endpoint titer of the last dilution exhibiting an optical density equal or greater than optical density measured for negative controls increased by one their standard deviation. FliD-specific IgA antibodies in extracts of gastrointestinal tracts, as well as in fecal extracts, were expressed in arbitrary units using a standard curve prepared with anti-FliD serum as described elsewhere [37].

### Isolation of Splenocytes

Mice were sacrificed, spleens were collected, and splenocytes isolated as previously described [16].

### Activation of Splenocytes

Splenocytes (2 × 10^5^/ml) were cultured in absence or presence of purified FliD for 48 h. Samples of supernatants containing released cytokines were collected and stored at − 80 °C.

### Measurement of Released Cytokines

Levels of IL-2, IL-4, IL-6, IL-10, IL-17A, IFN-γ, and TNF-α secreted by FliD-stimulated cells were measured using Cytometric Bead Array (CBA) Mouse Th1/Th2/Th17 Cytokine Kit (BD) kit as per the manufacturer’s protocol. Assays were performed using Accuri C6 Flow Cytometer (BD) and the results processed with system software. Six technical repeats were done for each animal in the group.

### Statistical Analysis

Results were statistically evaluated with one-way ANOVA and Tukey HSD test for post hoc analysis.

## Results

### Construction of Gene Fusions

**Fig. 1:**
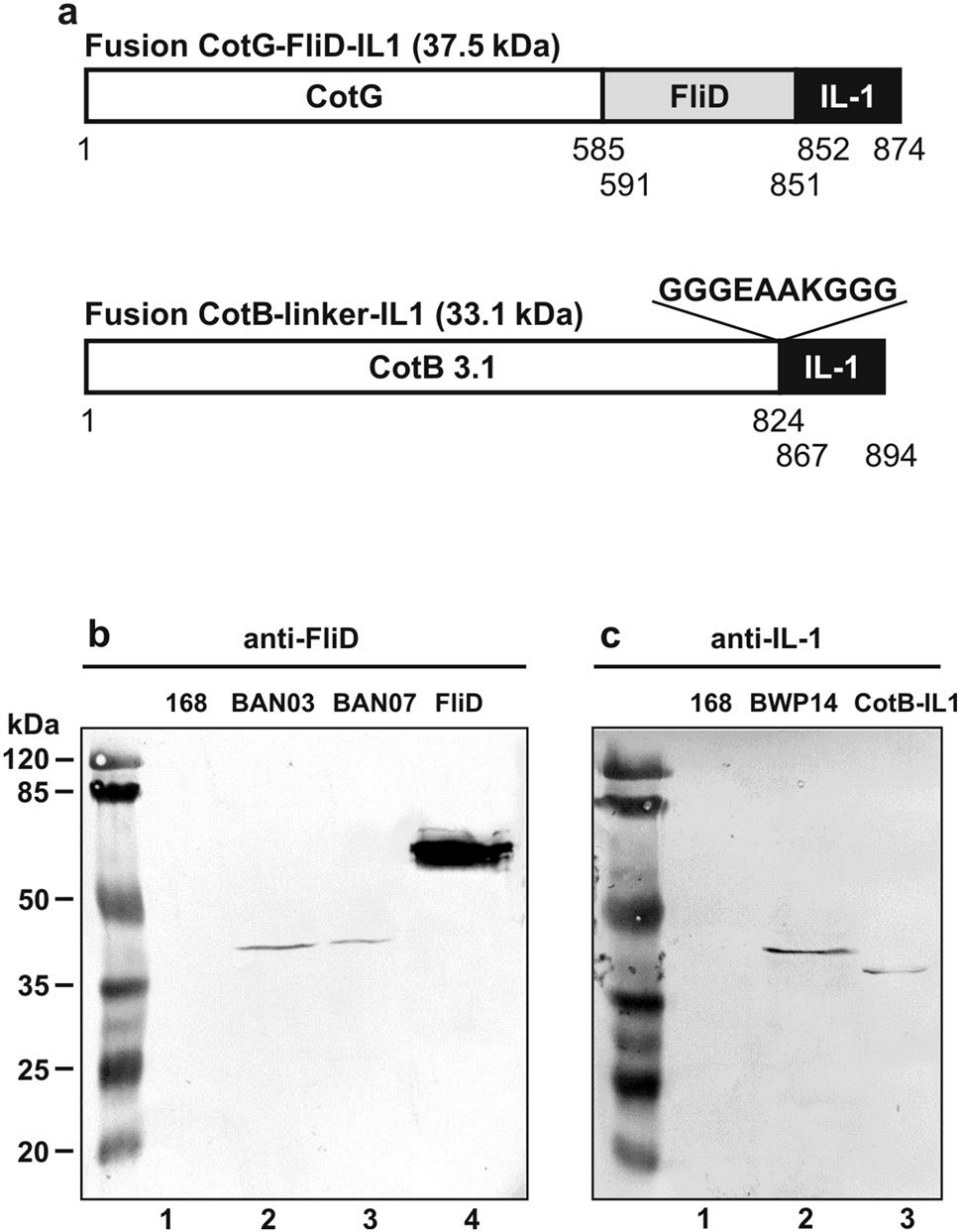
Western blot analysis of expression of the recombinant proteins. **a** Schematic representation of the two constructed fusion proteins. The calculated molecular masses of fusion proteins are shown in parentheses. Numbers under the schematic fusion proteins indicate nucleotide numbers. **b** Anti-FliD Western blotting. Lanes: 1, strain 168; 2, BAN03 (CotG-FliD); 3, BAN07 (CotG-FliD-IL1); 4, purified FliD (1 µg). **c** Anti-IL-1 Western blotting. Lanes: 1, strain 168; 2, BWP14 (CotB-IL1); 3, purified CotB-IL1 (1 µg). Spore coat proteins were extracted, fractionated on SDS-PAGE and analyzed by Western blotting as described in “Methods” section. Each lane contained 20 µg of total protein extract

To obtain recombinant spores presenting an antigen/adjuvant chimera protein, we selected a fragment of *C. difficile* FliD as antigen and VQGEESNDK peptide encompassing 163–171 amino acid residues of human IL-1β as adjuvant. The gene encoding FliD fragment/IL-1 peptide chimera was fused to the *cotG* spore coat protein, which retained its original promoter as in the BAN03 strain (CotG-FliD) [29]. The constructed strain was named BAN07 (CotG-FliD-IL1) and used for further analysis. Additionally, we have constructed a control recombinant strain BWP14 capable of expressing CotB protein fused with IL-1 fragment (VQGEESNDK) via a strong α-helical linker [38] to enhance its surface display (Fig. 1a). The C-terminus of CotB was truncated because it has been reported to confer genetic instability to fusion proteins containing the entire CotB protein [39]. Both recombinant strains and their isogenic parental strain 168 showed comparable sporulation and germination efficiencies (not shown).

### Spore Coat Expression

The localization of fusion proteins in the spore coat was tested by Western blotting with anti-FliD and anti-IL-1 fragment antisera. The anti-FliD antiserum recognized both, CotG-FliD and CotG-FliD-IL1 fusions in spore coat extracts, producing on Western blots two bands corresponding to estimated molecular weight of 42 kDa, with a slight shift of the band corresponding to CotG-FliD-IL1 fusion (BAN07) towards larger molecular weight (Fig. 1b). Since the calculated molecular weights of CotG-FliD and CotG-FliD-IL1 fusions are 36.6 kDa and 37.5 kDa, respectively, differences in positions of bands observed on Western blots were expected. Due to its electrophoretic properties, the CotG protein was reported to migrate in purified form as a 36 kDa protein instead of calculated 23.9 kDa [40]. Therefore, fusions prepared with the CotG as anchoring protein also exhibit discrepancies between the calculated and observed molecular weights. We were not able to detect IL-1 fragment displayed by our recombinant spores using commercial anti-IL-1 antibodies (not shown); therefore, we ourselves prepared mouse antiserum specific for it. For immunization of mice, we used VQGEESNDK peptide coupled with BSA protein as carrier. Western blot of both, the spore coat extract of the control strain BWP14 (CotB-IL1) and the purified CotB-IL1 fragment fusion protein, produced two bands corresponding to molecular weights 40 kDa and 38 kDa, respectively (Fig. 1c). The calculated molecular weight is 33.1 kDa for CotB-IL1 fusion protein in BWP14 spores and 34 kDa for CotB-IL-1 purified from *E. coli*. The difference between the calculated and observed molecular weights of purified CotB-IL-1 fusion most likely results from posttranslational processing of the CotB in *B. subtilis*, where it is phosphorylated by the CotH protein [41, 42]. Unexpectedly, anti-IL-1 antiserum did not recognize CotG-FliD-IL1 in the extract of BAN07 spore coat (not shown).

**Fig. 2:**
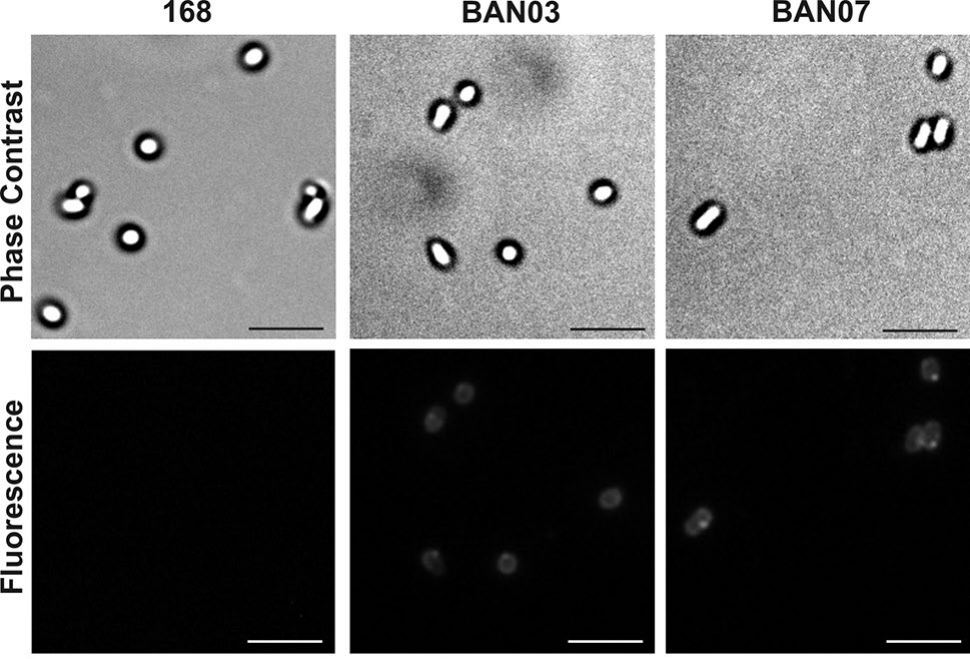
Spore surface display of CotG-fused FliD assessed by immunofluorescence microscopy. Purified, free spores of wild-type strain 168 and recombinant BAN03 and BAN07 were visualized by phase-contrast and immunofluorescence microscopy. The spores were incubated with mouse anti-FliD antibodies, followed by antimouse IgG–Cy3 conjugates. The same exposure time was used for all immunofluorescence images. Scale bar—10 µm

The presence of CotG-fused FliD fragments in the coats of the wild-type 168 and isogenic BAN03 and BAN07 spores was also analyzed by immunofluorescence microscopy with FliD-specific primary antibodies and antimouse IgG conjugated with Cy3. A fluorescent signal was observed for BAN03 spores, as previously reported [29], as well as for BAN07 (Fig. 2), indicating surface exposition of fusion proteins.

A quantitative determination of the amount of CotG-FliD-IL1 fusion protein in the coat of BAN07 spores was performed using a dot-blotting technique. Serial dilutions of purified FliD protein and coat proteins extracted from spores of the wild-type 168 and BAN07 recombinant strain and probed with anti-FliD antiserum and secondary antimouse IgG antibodies conjugated with alkaline phosphatase. Color products of reaction of alkaline phosphatase were analyzed by densitometry. The CotB-FliD-IL1 protein amounted to 0.07% of total coat proteins extracted from BAN07 spores which, regarding the concentration of total spore coat proteins estimated as 4.6 mg/ml, resulted in calculation of 199 CotG-FliD-IL1 molecules per single spore. The amount of CotG-FliD fusion protein in the coat of BAN03 spores, as analyzed in the same dot-blot experiment, was calculated to be 389 molecules per singe spore, with regard to total spore coat protein concentration in extracts estimated as 3.1 mg/ml.

### Immune Response to Recombinant Spores

**Fig. 3:**
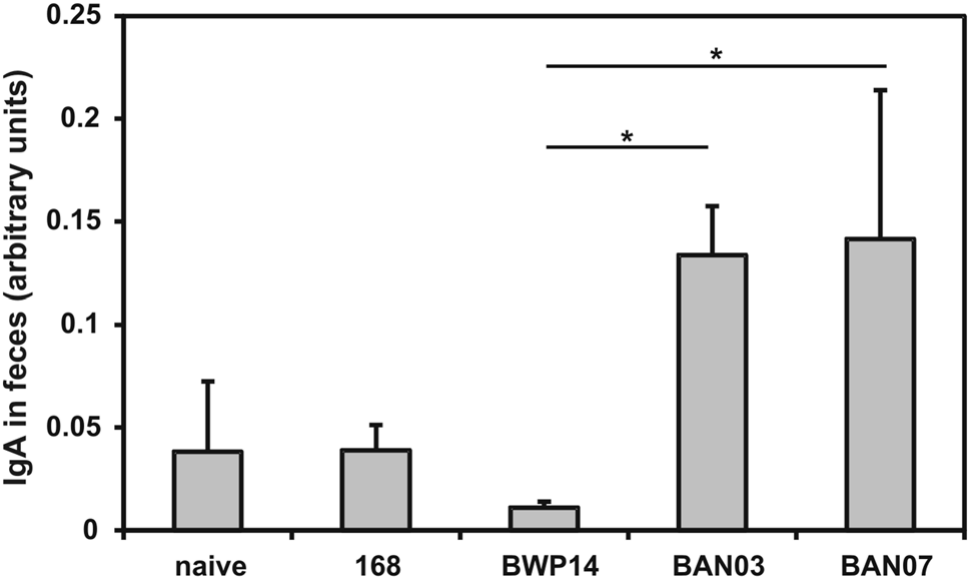
Level of FliD-specific IgA antibodies in feces collected from mice upon completion of immunization cycle (day 61). Mice were orally immunized with wild-type 168, recombinant BWP14 (CotB-IL1), BAN03 (CotG-FliD), or BAN07 (CotG-FliD-IL1) spores as described in “Methods” section. IgA levels expressed in arbitrary units calculated with anti-FliD antiserum standard curve. Each bar represents average of two independent experiments. Error bars represent standard deviation. *P < 0.05

Having prepared recombinant spores presenting the antigen/adjuvant chimeric protein (CotG-FliD-IL1) we used them for oral immunization of mice. Animals administrated with BAN03 spores received a total of 2.1 µg CotG-FliD protein (236.5 ng per dose) over the entire immunization cycle. Animals immunized with BAN07 spores received a total of 1.1 µg of CotG-FliD-IL1 chimeric protein (124 ng per dose). We observed no statistically significant increase in production of FliD-specific IgG antibodies in sera and IgA antibodies in gastrointestinal tracts of immunized animals (not shown). We were able to detect increased levels of FliD-specific IgA antibodies in feces collected from animals immunized with either BAN03 (CotG-FliD) or BAN07 (CotG-FliD-IL1) spores (Fig. 3). Observed increase was not statistically significant when tested against naïve or the group immunized with empty 168 spores (*P* = 0.0932), but showed statistical significance against the group immunized with BWP14 spores displaying CotB-IL1 fusion (*P* = 0.0104).

### IL-1 Fragment Modulates Immune Response Elicited by Recombinant Spores

**Fig. 4:**
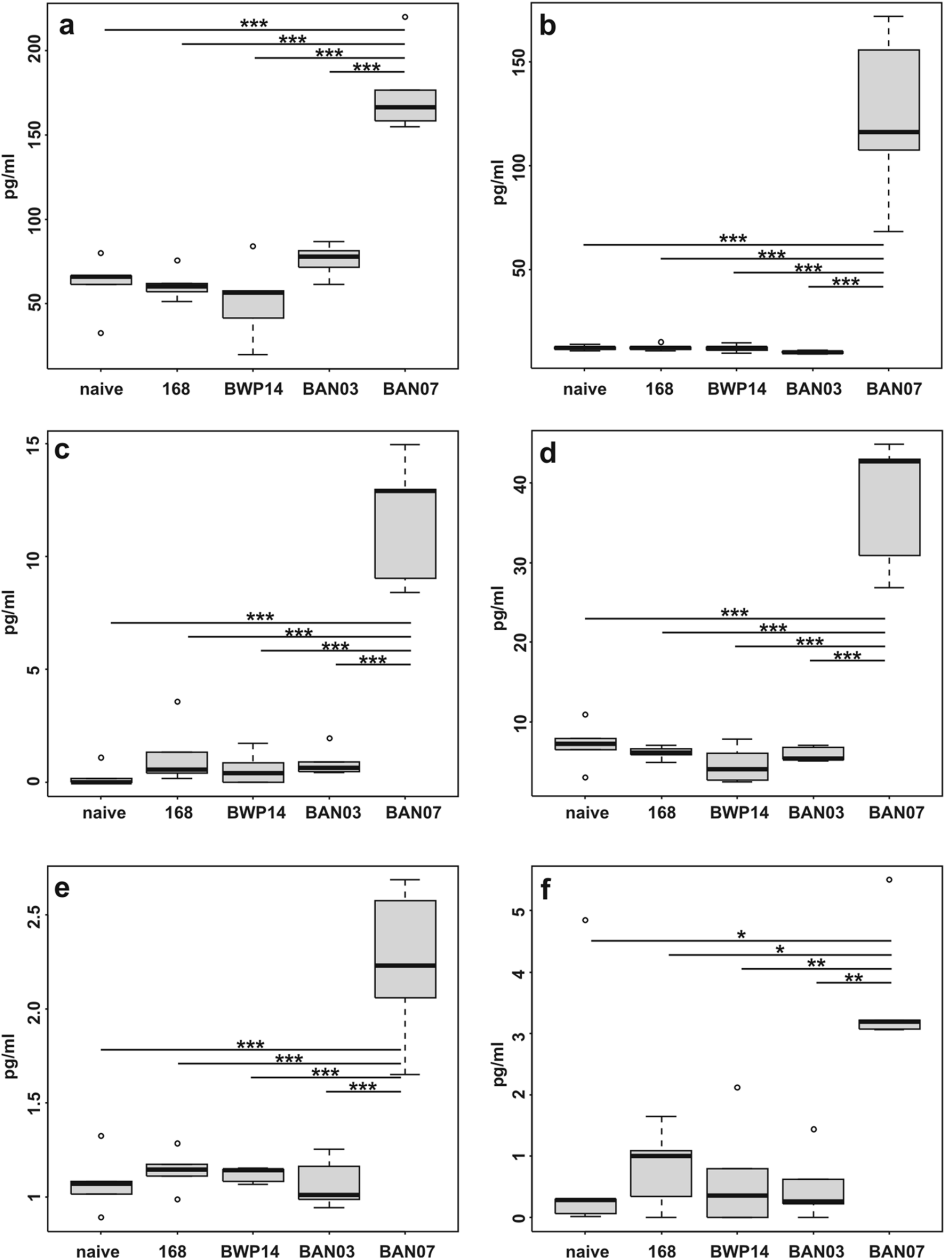
Characterization of the immune response. Groups (*n* = 5) of BALB/c mice were orally immunized with wild-type 168, recombinant BWP14 (CotB-IL1), BAN03 (CotG-FliD), or BAN07 (CotG-FliD-IL1) spores as described in “Methods” section. Isolated splenocytes were stimulated with purified FliD, and levels of following cytokines were measured in cell culture supernatants: **a** TNF-α, **b** IFN-γ, **c** IL-17A, **d** IL-6, **e** IL-4, **f** IL-2. Each plot presents averages of two independent experiments. Statistical analysis performed as described in “Methods” section. *P < 0.05; **P < 0.01; ***P < 0.001

Since IL-1 was shown to possess adjuvant activity in mucosal immunizations and be capable of inducing an antibody response and cell-mediated immunity against bacterial antigens [43], we wanted to verify, whether the presence of IL-1 fragment in spore-displayed chimeric protein influenced polarization of elicited immune response. We characterized the immune response elicited by FliD-presenting spores by analyzing profiles of cytokines secreted by FliD-stimulated splenocytes isolated from the immunized animals. We observed increases in production of IL-2, IL-4, IL-6, IL-17A, TNF-α, and IFN-γ in the group of mice immunized with BAN07 spores (CotG-FliD-IL1) (Fig. 4). No statistically significant increase in secretion of analyzed cytokines was observed for any other group of animals in the study. The obtained results clearly suggest immunomodulatory action of IL-1 fragment present in CotG-FliD-IL1 chimeric protein displayed by BAN07 spores.

## Discussion

Mucosal vaccines, despite undoubted benefits of their use, suffer from several problems limiting their usefulness in the real world. Two major drawbacks are the stability of antigens on mucosal surfaces and the selection of appropriate mucosal adjuvant [7]. In this study, we proposed to use for such application *B. subtilis* spores displaying a chimeric antigen/adjuvant protein. Due to such an approach, we could benefit from the spore display technology which was shown in many applications to protect a passenger protein displayed on the spores’ surface against different environmental conditions.

The choice of antigen, a fragment of flagellar cap FliD protein of *C. difficile*, was dictated by the fact that we have already used it in our previous studies where we have prepared recombinant spores presenting fragment of this protein in fusion with different spore coat proteins [31] and analyzed their interaction with the immune system [27]. Moreover, we have also used the entire FliD protein to present it on the spore surface using adsorption technique and were able to elicit immune response in mucosally immunized mice when IL-2-presenting recombinant spores were used as antigen carriers [18]. As the adjuvant part of the chimeric protein, we selected human IL-1β fragment 163–171 (VQGEESNDK), because of its strong adjuvanticity and lack of pro-inflammatory properties [24]. Moreover, oral immunizations with recombinant *Lactobacillus casei* secreting IL-1β have proved adjuvant activity of this molecule for intragastric immunizations [44].

In our CotG-FliD-IL1 construct, we encountered problems with recognition of IL-1 part by anti-IL-1 antisera, which most probably resulted from properties of this fusion protein. The IL-1β fragment was attached in both, BWP14 (CotB-IL1) spores and purified CotB-IL1 fusion protein, via a strong α-helical linker [38], which most probably enhanced its recognition by anti-IL-1 antiserum (Fig. 2b). Such linker was not used in construction of BAN07 recombinant spores where IL-1 fragment was directly attached to CotG-FliD fusion protein. Moreover, CotG-anchored fusion proteins were also speculated to undergo a proteolytic cleavage [35]. Nonetheless, we were able to detect CotG-FliD-IL1 fusion protein using anti-FliD antiserum. Verification of the presence of entire *cotG-FliD-IL1* fusion gene in the BAN07 chromosome (Fig. S1) as well as shift in molecular weight of the band detected by anti-FliD antiserum in BAN07 spore coat extracts (Fig. 1b) clearly suggested that CotG-FliD-IL1 protein was present in the coat of spores produced by this strain.

As is well known, the efficiency of display of a heterologous protein on the surface of recombinant spores will depend on properties of an individual fusion. Therefore, we were not surprised to observe different number of estimated CotG-FliD-IL1 molecules (199) per single spore, extracted from BAN07 spores, compared to 389 molecules/spore of CotG-FliD in the coat of BAN03 spores. The reported amount of CotG-FliD fusion proteins is lower than that previously reported [29]; nevertheless, the observed difference might result from different conditions used for production of spores, e.g., medium composition, volume of the culture, intensity of agitation, as well as the different efficiency of spore coat proteins’ extraction.

Despite the observed twofold lower amount of FliD fragment molecules displayed by BAN07 spores compared to BAN03, levels of FliD-specific IgA antibodies in feces of animal immunized with both spores were similar. It is important to note that, while the IL-1β synthetic fragment 163–171 (VQGEESNDK) has been used as adjuvant for parenteral immunizations [20], the entire IL-1 has been reported to function as adjuvant in oral immunizations, preventing development of mucosal tolerance to delivered antigen [45]. Regarding low levels of FliD-specific IgA antibodies detected in feces of immunized animals, the statement that we are clearly observing development of the humoral response to administrated antigen should be made with caution. Nonetheless, the presence of IL-1 fragment in BAN07 spores evidently influences development of the cellular immune response to spore-delivered FliD fragment. Lack of increase in secretion of analyzed cytokines in the case of animals immunized with BAN03 (CotG-FliD) spores is in line with results of our previous studies, in which we have shown that intranasal administration of these spores neither led to production of FliD-specific antibodies nor resulted in the development of cell-mediated immunity [27]. Therefore, the observed increase in the production of these cytokines in the case of animals administrated with BAN07 spores (GotG-FliD-IL1) clearly indicates the immunomodulatory action of IL-1 fragment present in the displayed protein fusion. It is important to emphasize here that this cytokine has been shown to be an effective adjuvant for mucosal and systemic immune responses [46]. The entire IL-1β enhances antigen-driven expansion and differentiation of CD4 T-cells [47], as well as antigen-driven responses of both CD8 and CD4 T-cells [48]. This cytokine was also shown to stimulate proliferation of T-cells [49, 50].

To summarize, in this study, we have proven that it is possible to successfully display a chimeric antigen/adjuvant protein on the coat of recombinant spores. Moreover, we have shown that such spores elicited immune response, which was modulated by the presence of IL-1β fragment (VQGEESNDK). Taken together, our results suggest that this peptide can be used as adjuvant in mucosal immunizations containing spores of *B. subtilis*.

## Electronic supplementary material

Below is the link to the electronic supplementary material.

Supplementary material 1 (TIF 336 KB)
